# Regional variation in total flavonoid content and antioxidant activity of *Artemisia dracunculus* L. in Xinjiang, China

**DOI:** 10.1186/s12870-026-09519-1

**Published:** 2026-07-17

**Authors:** Yu Zhang, Yingxuan Geng, Xiaoli Ma, Zhangjian Huang, Hailiqian Taoerdahong, Junmin Chang

**Affiliations:** 1 Key Laboratory of Active Components of Xinjiang Natural Medicine and Drug Release Technology, No. 567 Shangde North Road, Shuimogou District, Urumqi, Xinjiang Uygur Autonomous Region 830011 China; 2 College of Pharmacy, Xinjiang Medical University, No. 567 Shangde North Road, Shuimogou District, Urumqi, Xinjiang Uygur Autonomous Region 830011 China; 3 College of Basic Medical Sciences, Xinjiang Medical University, Urumqi, Xinjiang Uygur Autonomous Region 830011 China; 4 College of Traditional Chinese Medicine, Xinjiang Medical University,, No. 567 Shangde North Road, Shuimogou District, Urumqi, Xinjiang Uygur Autonomous Region 830011 China

**Keywords:** Antioxidant activity, *A. dracunculus*, Total flavonoids

## Abstract

**Background:**

*Artemisia dracunculus* L. *(A. dracunculus)* is a distinctive plant resource in Xinjiang, possesses both medicinal and culinary value, with distinct regional characteristics.

**Methods:**

In this study, total flavonoids extracted from *A. dracunculus* (TFAD) were obtained using a reflux method, with extraction parameters optimized through single-factor experimental design. Ultraviolet-visible spectrophotometry was used for both qualitative and quantitative assessment of TFAD. Samples collected from Jimusaer, Hami, and Tacheng in Xinjiang were analyzed for total flavonoid content and antioxidant activity.

**Results:**

The optimized extraction conditions included 50% ethanol as the solvent, a solid-to-liquid ratio of 1:30, an extraction temperature of 80 °C, and a duration of 8 h. Under these conditions, the TFAD content in samples from Jimusaer, Hami, and Tacheng was 107.0146 ± 1.4986, 111.9548 ± 1.6136, and 124.8996 ± 1.3801 mg g⁻¹, respectively. Statistically significant regional differences were observed (*p* < 0.01), with the highest flavonoid concentration found in samples from Tacheng. Antioxidant activities of TFAD from the three regions were assessed using in vitro free radical scavenging assays, with ascorbic acid (AA) serving as the positive control. TFAD extracted from Jimusaer exhibited the strongest antioxidant capacity, with IC₅₀ values of 31.31 ± 1.19 and 114.04 ± 5.56 µg mL⁻¹ against DPPH and ABTS⁺· radicals, respectively, compared with 7.25 ± 0.56 and 154.18 ± 9.54 µg mL⁻¹ for AA.

**Conclusion:**

These results suggest that TFAD from the Jimusaer region demonstrates superior antioxidant activity, providing a basis for further exploration and potential utilization of *A. dracunculus* as a functional resource in Xinjiang.

## Introduction

Oxidative stress is a pathological condition marked by an imbalance between the generation of endogenous reactive oxygen species and the capacity of the antioxidant defense system. It has been recognized as a key contributor to the initiation and progression of various diseases [1]. Elevated levels of free radicals can result in oxidative damage to cellular components, including DNA, lipid membranes, and proteins, subsequently initiating inflammatory responses, genetic mutations, apoptosis, and functional decline in affected tissues. These pathological processes constitute major etiological mechanisms across a wide range of disorders and critically amplify disease progression. In certain conditions, oxidative stress is further intensified, perpetuating a self-reinforcing cycle that significantly compromises human health [2]. Along with the increasing emphasis on natural and health-promoting interventions, naturally derived antioxidant compounds, particularly polyphenols, flavonoids, and carotenoids sourced from foods and agricultural by-products, have demonstrated substantial potential for application in food science, nutrition, pharmacology, and cosmetic science. This potential is attributed to their strong antioxidant properties, favorable biocompatibility, and low toxicity profiles [3].

*Artemisia dracunculus* L. *(A. dracunculus)*, commonly referred to as tarragon, is a species with a longstanding role in traditional Asian medicine. It is primarily distributed across Iran, Pakistan, Azerbaijan, India, and neighboring regions, and is present in northern and northwestern China [4]. In Xinjiang, wild populations of *A. dracunculus* are widespread, particularly in grasslands and semi-desert steppes along the northern piedmont of the Tianshan Mountains in northern Xinjiang, and in similar habitats along the southern slopes in southern Xinjiang. The species is commonly found in dry valleys, river terraces, and grasslands at elevations ranging from 500 to 2,500 m, and demonstrates notable tolerance to drought, salinity, cold temperatures, and nutrient-poor soils. Recognized for its characteristic aroma, which resembles that of Sichuan pepper and mint, *A. dracunculus* is utilized both as a medicinal and culinary herb, valued for its distinctive flavor profile [5]. In several European countries, it is widely employed as a culinary ingredient and is often referred to as the “king of spices” [6]. It is traditionally used for the management of constipation, intestinal spasms, and peptic ulcers [7]. In various Asian regions, the whole plant is applied in traditional medicine for the treatment of wind-cold syndrome, gastrointestinal disturbances, and allergic skin conditions, and has been reported to exhibit anti-inflammatory, hemostatic, and analgesic properties [4, 8].

Although *A. dracunculus* has a long history of use in traditional medicine, research over the past decade has primarily focused on its specific bioactive constituents and the mechanisms underlying its pharmacological effects in disease prevention and management. Findings from recent studies have substantiated the traditional applications of *A. dracunculus* while uncovering additional pharmacological effects. Essential oils, aqueous extracts, and alcoholic extracts of *A. dracunculus* have demonstrated a range of bioactivities, including antimicrobial, antioxidant, immunomodulatory, antitumor, hepatoprotective, hypoglycemic, antidepressant, neuroprotective, and antiparasitic effects [9–23].

In 2018, Zarezade et al. assessed the antioxidant and hepatoprotective effects of alcoholic extracts derived from the aerial parts of *A. dracunculus* in a rat model of CCl^₄−^induced hepatotoxicity. The extract exhibited significant antioxidant activity, as determined by ferric reducing antioxidant power (FRAP), 2,2-diphenyl-1-picrylhydrazyl (DPPH), and 2,2′-azino-bis(3-ethylbenzothiazoline-6-sulfonic acid) radical cation (ABTS⁺·) radical scavenging assays, and was associated with low toxicity. Histopathological analysis confirmed its protective effects against CCl^₄−^induced liver injury, with the proposed mechanism involving attenuation of oxidative stress [17]. In 2024, Țicolea and co‑workers investigated the antioxidant and anti‑inflammatory activities of *A. dracunculus* extract using in vivo experiments in rats. The results demonstrated that the extract exerted anti‑inflammatory effects by downregulating the levels of inflammatory mediators including NF-κB p65 and IL − 1β. Meanwhile, it modulated the total oxidative status and total antioxidant capacity of the organism, thereby alleviating oxidative stress injury. Key toxicity indicators such as serum transaminases and creatinine in rats remained within normal ranges throughout the experiment, verifying that the extract caused no obvious hepatotoxicity or nephrotoxicity. This finding indirectly supports the low‑toxicity profile of flavonoids and other bioactive constituents present in *A. dracunculus* [24]. These findings support the classification of *A. dracunculus* as a high-potential, safe and low-toxic candidate for development in the “medicine-food homology” sector.

Flavonoids represent a principal class of bioactive constituents in *A. dracunculus*. Following purification using AB-8 macroporous resin, the total flavonoid content in dried extracts from wild *A. dracunculus* has been reported to reach 52.4% [25]. The Xinjiang region offers highly favorable geographical and climatic conditions such as high solar irradiance, substantial diurnal temperature fluctuations, and prolonged growing seasons, which are conducive to the accumulation of bioactive compounds. These environmental factors suggest that *A. dracunculus* grown in Xinjiang may contain elevated levels of flavonoids and exhibit enhanced bioactivity. However, due to the extensive land area and considerable variability in longitude, latitude, and ecological conditions, fluctuations in the concentration and distribution of plant-derived bioactive compounds are likely.

In this study, reflux extraction was used to isolate total flavonoids extracted from *A. dracunculus* (TFAD) samples collected from three locations within Xinjiang: Jimusaer, Hami, and Tacheng. The extraction process was subsequently optimized. Ultraviolet-visible (UV-Vis) spectrophotometry was used for both qualitative and quantitative analysis of TFAD, and the flavonoid content among regional samples was compared. Ascorbic acid (AA) served as the positive control. The Asymptotic1 model was applied to fit the dose-response relationships between TFAD concentrations and the radical scavenging rates of DPPH and ABTS⁺·, and IC₅₀ values were calculated to assess the antioxidant activities of TFAD from the different regions within Xinjiang.

## Materials and methods

### Samples and reagents

#### *A. dracunculus* samples

All samples of *A. dracunculus* were collected in May 2024 from river terrace habitats located in the 164th Regiment in Tacheng, Jimusaer County in Changji Prefecture, and Yiwu County in Hami Prefecture, Xinjiang. Botanical identification was conducted by Professor Xinling Wang of the Department of Natural Medicines and Pharmacognosy, School of Pharmacy, Xinjiang Medical University. The upper aerial portions extending 8–10 cm from the plant apex were harvested. The collected plant materials were shade-dried, ground, and then passed through a 100-mesh sieve. The resulting powder was used for subsequent experimental procedures.

#### Reagents

Rutin reference standard (Batch No. B20771) and DPPH (Batch No. KS343393) were obtained from Shanghai Yuanye Bio-Technology Co., Ltd. ABTS diammonium salt (Batch No. C17275878) was obtained from Shanghai Macklin Biochemical Technology Co., Ltd. Potassium persulfate, AA, and sodium hydroxide were supplied by Shanghai Aladdin Biochemical Technology Co., Ltd. Anhydrous ethanol was obtained from Lianlong Bohua (Tianjin) Pharmaceutical Chemistry Co., Ltd. Aluminum nitrate, petroleum ether, and sodium nitrite were purchased from Tianjin Xinbote Chemical Co., Ltd.

### Experimental methods

#### Preparation of total flavonoids from *A. dracunculus*

An appropriate quantity of *A. dracunculus* powder was combined with 95% ethanol at a mass-to-volume ratio of 1:3 (g : mL). The mixture was subjected to reflux extraction in a water bath at 60 °C for 2 h and subsequently filtered to collect the plant residue. The residue was air-dried at room temperature in a cool, ventilated environment to yield defatted plant material for further extraction. A 2.00 g portion of the dried material was accurately weighed and mixed with 50% ethanol at a solid-to-liquid ratio of 1:30 (g : mL). The mixture was refluxed at 80 °C for 8 h and then filtered to obtain the filtrate. Petroleum ether was added to the filtrate at a volume ratio of 2:1 (petroleum ether : filtrate), and liquid-liquid partitioning was performed for 2 h to remove lipophilic substances. Following removal of the petroleum ether layer, the remaining solution was concentrated using a rotary evaporator at 55 °C under − 0.1 MPa and 120 rpm until approximately 5 mL remained. The resulting concentrate was transferred to a 50 mL centrifuge tube, and anhydrous ethanol was added at a volume ratio of 4:1 (anhydrous ethanol : sample). The mixture was stored at 4 °C for 24 h. After standing, the solution was centrifuged at 2,000 rpm for 10 min. The supernatant was collected and further concentrated to approximately 5 mL under the same evaporation conditions. Finally, the volume was adjusted to 25 mL with 60% ethanol in a volumetric flask to obtain the TFAD extract.

#### Determination of total flavonoids content from *A. dracunculus*

Aluminum chloride colorimetric reaction: Aliquots of 2.0 mL of 60% ethanol and 2.0 mL of TFAD extract were separately placed into 10 mL stoppered test tubes and designated as control solution A and sample solution B, respectively. To each tube, 1.0 mL of 5% AlCl₃ solution was added. After standing for 5 min, color changes in both solutions were visually assessed. Subsequently, both solutions were exposed to ultraviolet light, and fluorescence was observed.

UV-Vis spectrophotometry: A 0.2 mL aliquot of TFAD extract was accurately transferred into a 25 mL volumetric flask and diluted to volume with 60% ethanol. The mixture was shaken and allowed to stand for 2 min. The ultraviolet absorption spectrum was then recorded using a UV-2700 UV-Vis spectrophotometer. The scanning range was set from 200 to 600 nm, with a scanning speed of 120 nm min⁻¹ and a slit width of 1.0 nm.

#### Quantification of TFAD

Preparation of standard curve: Rutin (5 mg) was dissolved in 60% ethanol and diluted to 25 mL to prepare a stock solution. Aliquots of 0, 1, 2, 3, 4, 5, and 6 mL of the stock solution were each diluted to 25 mL with 60% ethanol to obtain rutin solutions of varying concentrations. From each solution, a 1.0 mL aliquot was transferred into a 25 mL volumetric flask. Subsequently, 1.0 mL of 5% (w/v) NaNO₂ solution was added, and the mixture was shaken and allowed to react for 6 min. Then, 1.0 mL of 10% (w/v) Al(NO₃)₃ solution was added, followed by a second 6-minute reaction. Finally, 1.0 mL of 4% (w/v) NaOH solution was added, and each solution was diluted to 25 mL with 60% ethanol and allowed to stand for 15 min. Absorbance (A) was measured at 510 nm. A standard calibration curve was constructed using rutin concentration (C) as the abscissa and absorbance (A) as the ordinate. The resulting linear regression equation was A = 9.3305 C + 0.0608, with a correlation coefficient (R²) of 0.9999.

Determination of TFAD content: A 1.0 mL aliquot of TFAD extract was accurately transferred into a 25 mL volumetric flask and diluted with 5 mL of 60% ethanol. Subsequently, 1.0 mL of 5% NaNO₂ solution was added, and the mixture was allowed to stand for 6 min. This was followed by the addition of 1.0 mL of 10% Al(NO₃)₃ solution, and the solution was again allowed to stand for 6 min. Then, 10 mL of 4% NaOH solution was added, and the mixture was made up to 25 mL with 60% ethanol and allowed to stand for 15 min. Absorbance (A) was recorded at 510 nm. The TFAD concentration (C) in the sample solution was calculated using the established regression equation. The TFAD yield was determined using the following formula: *R = C × V × D / m*, where R is the TFAD yield (mg g^− 1^), C is the concentration of the target in the final diluted solution (mg mL^− 1^), V is the final volumetric flask volume after dilution (25 mL), D is a dimensionless dilution factor(25), and m is the mass of the *A. dracunculus* sample (2 g).

#### Evaluation of antioxidant activity of TFAD

The scavenging effect of total flavonoids from *A. dracunculus* on ABTS^+^· was tested according to the method reported by Alshehri et al. [26] with slight modifications: A 7 mM ABTS solution and a 2.45 mM potassium persulfate solution were prepared using 60% ethanol. Equal volumes of the two solutions were mixed and stored at 4 °C in the dark for 12 h. The resulting solution was diluted with 60% ethanol until an absorbance of approximately 0.7 at 734 nm was achieved to obtain the ABTS working solution. The TFAD extract was diluted with 60% ethanol to prepare test solutions at defined concentration ranges and stored at 4 °C for 12 h prior to use.

A 2.0 mL aliquot of ABTS working solution was mixed with 100 µL of TFAD solution at each concentration and incubated in the dark at 4 °C for 30 min. The absorbance was measured at 734 nm and recorded as A₁. A mixture of 2.0 mL of 60% ethanol and 100 µL of TFAD solution was prepared and measured under the same conditions, with the absorbance recorded as A₂. A mixture of 2.0 mL of ABTS working solution and 100 µL of 60% ethanol was measured under the same conditions and recorded as A₀. All absorbance values were determined using 60% ethanol as the blank. Each measurement was conducted five times, with the highest and lowest values discarded and the mean of the remaining three values was used for analysis. The ABTS⁺·scavenging rate was calculated using the following equation:

$$\mathrm{Surviving}\hspace{0.17em}\mathrm{rate}\hspace{0.17em}(\%):=\:[1\:-\:({\mathrm{A}}_{1}\hspace{0.17em}-\hspace{0.17em}{\mathrm{A}}_{2})\:/\:{\mathrm{A}}_{0}]\:\times\:\:100\%$$

The scavenging rate of total flavonoids from *A. dracunculus* on DPPH· was tested using a similar method as described above: A 0.1 mM DPPH solution was prepared using 60% ethanol and stored at 4 °C in the dark until use. The TFAD extract was diluted with 60% ethanol to prepare test solutions at a range of concentrations, optimized for measurement within the 20–80% DPPH· scavenging range. For each concentration, 2 mL of TFAD solution was mixed with 2 mL of DPPH solution and incubated in the dark for 30 min. The absorbance was measured at 517 nm and recorded as A₁. A mixture of 2 mL of 60% ethanol and 2 mL of TFAD solution was measured and recorded as A₂. A mixture of 2 mL of DPPH solution and 2 mL of 60% ethanol was measured and recorded as A₀. All absorbance readings were conducted using 60% ethanol as the blank. Measurements were repeated five times, with the highest and lowest values excluded, and the mean of the remaining three values was used for analysis. The DPPH scavenging rate was calculated using the same formula as that used in the ABTS⁺·assay.

#### Calculation and fitting of half-maximal inhibitory concentration (IC_50_)

The scavenging activity of DPPH· and ABTS^+^·were measured according to the method described above. The dose–response curves were fitted using the five-parameter logistic model:

$$\:\mathrm{y}\hspace{0.17em}=\hspace{0.17em}{\mathrm{A}}_{\mathrm{m}\mathrm{i}\mathrm{n}}\:+\:({\mathrm{A}}_{\mathrm{m}\mathrm{a}\mathrm{x}}\:-\:{\mathrm{A}}_{\mathrm{m}\mathrm{i}\mathrm{n}})\:/\:{[1\:-\:{(\mathrm{x}\:0\:/\:\mathrm{x})}^{\mathrm{h}}]}^{\mathrm{s}}$$

where y is the radical scavenging rate (%), *x* is the sample concentration, A _min_ and A _max_ represent the minimum and maximum response values, *x*_0_ is the half-maximal inhibitory concentration (IC_50_), *h* is the Hill slope and *s* is asymmetry parameter. The original fitting was performed using the five-parameter logistic model with the asymmetry parameter *s* fixed at 1, which is mathematically equivalent to the standard four-parameter logistic model (4PL). Curve fitting and IC_50_ calculation were performed using OriginPro 2024b (OriginLab Corporation, Northampton, MA, USA).

### Statistical analysis

Statistical analysis was performed using OriginPro 2024b (OriginLab Corporation, Northampton, MA, USA). One-way ANOVA was applied to compare the differences in total flavonoid content among Jimusaer, Hami, and Tacheng regions. The Shapiro-Wilk test was used to assess the normality of the data, and Levene’s test was performed to verify the homogeneity of variances. Tukey’s HSD test was conducted for post-hoc multiple comparisons. Differences were considered statistically significant when *P* < 0.05.

## Results and discussion

### Identification of TFAD

Flavonoids react with aluminum chloride to produce a characteristic yellow, yellow-green, or orange-yellow coloration under natural light, with the specific hue dependent on the flavonoid structure. The phenolic hydroxyl groups of flavonoids can chelate Al³⁺ ions to form stable aluminum complexes, which exhibit enhanced or distinct fluorescence under ultraviolet illumination. This reaction is recognized as a specific method for the identification of flavonoids [27].

**Fig. 1:**
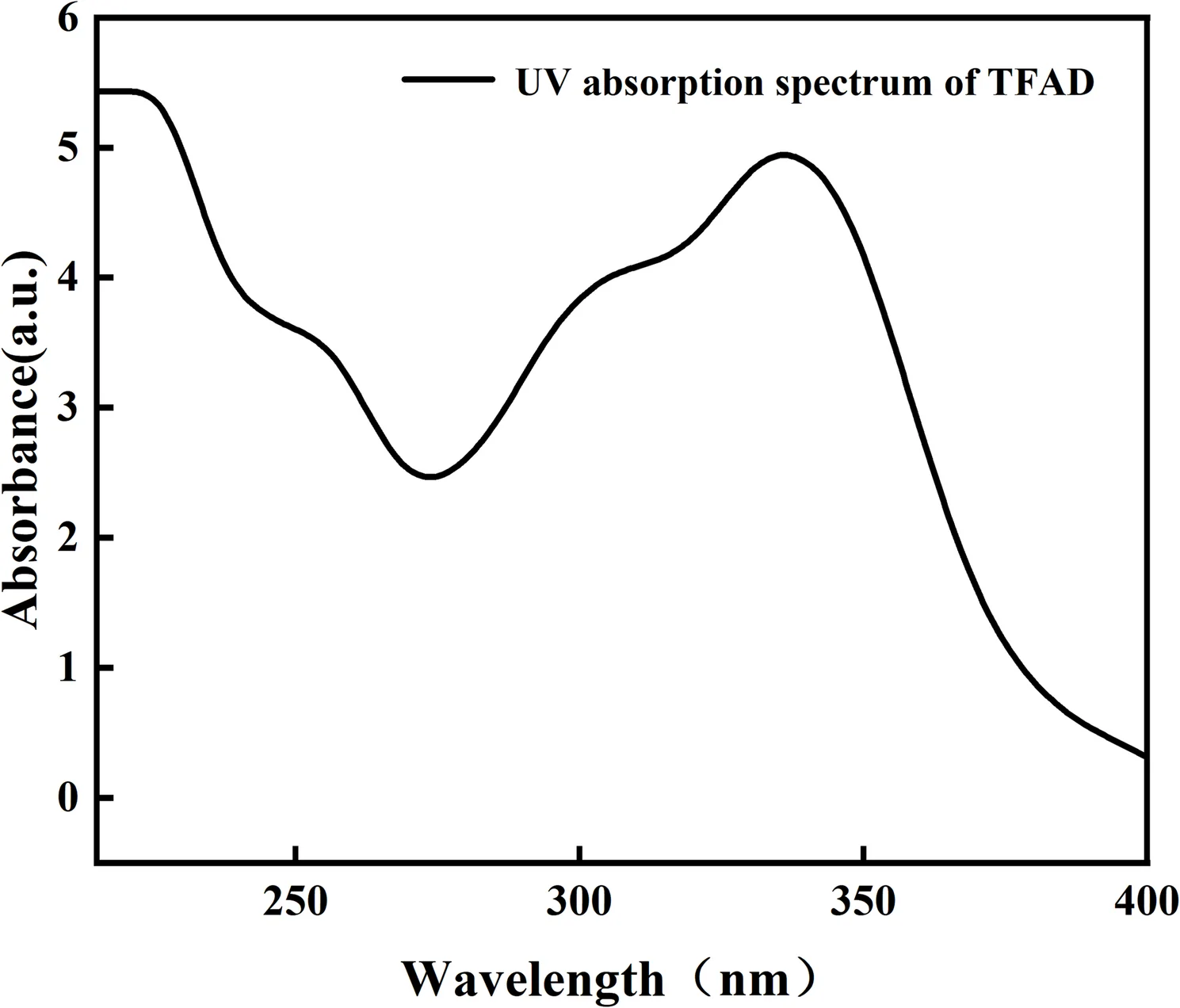
UV-Vis absorption spectrum of the TFAD. UV-Vis: Ultraviolet-visible; TFAD: total flavonoids extracted from *A. dracunculus*

In this study, the TFAD extract developed a yellow coloration following reaction with aluminum chloride and exhibited yellow fluorescence under ultraviolet light, indicating the presence of flavonoid compounds. UV-Vis spectrophotometric analysis was conducted over a wavelength range of 200–600 nm, and the resulting spectrum is presented in Fig. 1. A prominent absorption peak was detected at 250 nm, corresponding to Band II (220–280 nm), which is associated with electronic transitions in the A-ring of the flavonoid nucleus and characteristic of the benzoyl system. A second strong absorption peak was observed near 350 nm, corresponding to Band I (300–400 nm), which arises from electronic transitions in the cinnamoyl system of the B-ring [28]. The presence of these two characteristic absorption bands provides further confirmation of flavonoid compounds in the TFAD extract.

### Optimization of the TFAD extraction process

**Fig. 2:**
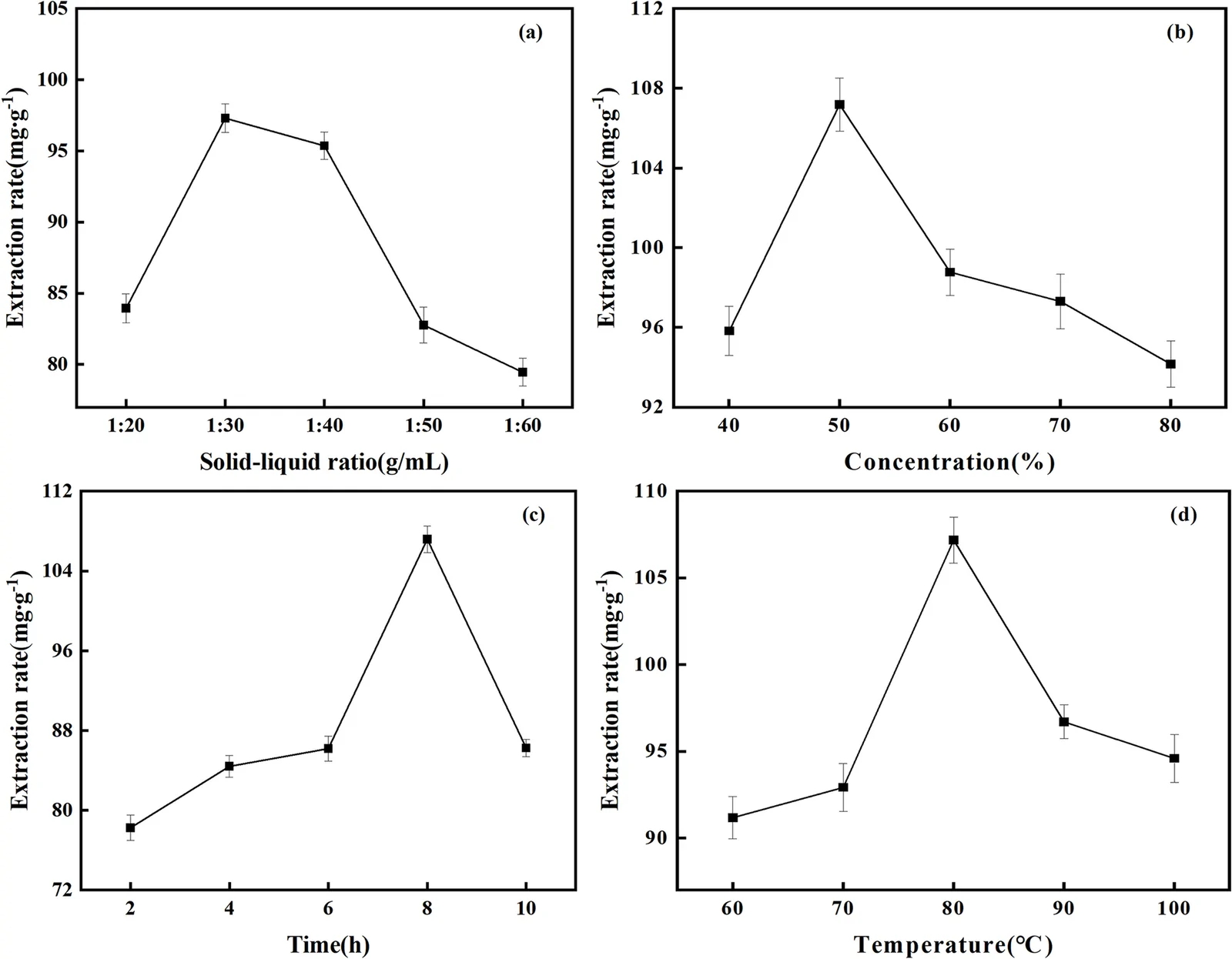
Effects of extraction parameters on TFAD yield: **a** solid-to-liquid ratio; **b** ethanol concentration; **c** extraction time; **d** extraction temperature. TFAD: total flavonoids extracted from *A. dracunculus*

Following validation of the extraction protocol, the process was further optimized using TFAD yield as the evaluation index. The influence of four variables: solid-to-liquid ratio, ethanol concentration (v/v), extraction time, and extraction temperature on TFAD yield was systematically assessed. The results are presented in Fig. 2.

Figure 2(a) shows the comparison of the TFAD obtained under different solid-to-liquid ratios when the solvent is 70% ethanol, the temperature is 80℃, and the extraction time is 8 h. As presented in Fig. 2(a), the TFAD yield reached a maximum of 97.3019 ± 0.9925 mg g⁻¹ at a solid-to-liquid ratio of 1:30. With increasing ratio, the yield initially increased and subsequently declined. This trend may be attributable to the limited solvent volume at lower ratios, which may hinder complete dissolution of flavonoids, while excessive solvent volumes can reduce extraction efficiency. Therefore, under otherwise constant conditions, the optimal solid-to-liquid ratio was determined to be 1:30.

Figure 2(b) illustrates the influence of different ethanol volume fractions on the extraction rate of TFAD when the extraction temperature is 80 °C and the extraction time is 8 h. A maximum yield of 107.1821 ± 1.3270 mg g⁻¹ was observed at 50% ethanol. Extraction efficiency decreased at both higher and lower ethanol concentrations. This behavior is likely due to the influence of ethanol concentration on solvent polarity. A high solvent polarity may be unfavorable for TFAD dissolution, whereas a low polarity may enhance the solubility of lipophilic impurities, causing early solvent saturation and decreased TFAD recovery.

As presented in Fig. 2(c), TFAD yield increased progressively with longer extraction durations and reached a peak of 107.1821 ± 1.3270 mg g⁻¹ at 8 h. Prolongation beyond 8 h resulted in a decline in yield. This reduction may be due to under-extraction at shorter durations and potential oxidative degradation, hydrolysis, or thermal decomposition of flavonoids at extended durations, resulting in loss of TFAD.

Figure 2(d) presents the effect of extraction temperature on TFAD yield, which followed a unimodal trend. At lower temperatures, reduced cell membrane permeability, slower solvent dynamics, and lower mass transfer rates may limit the dissolution and diffusion of flavonoids. Conversely, excessively high temperatures may promote thermal oxidation and degradation of flavonoids, along with enhanced extraction of proteins, pigments, and other impurities, potentially decreasing TFAD solubility.

In conclusion, the optimal extraction conditions for TFAD were identified as follows: a solid-to-liquid ratio of 1:30, an ethanol concentration of 50%, an extraction time of 8 h, and an extraction temperature of 80 °C. The maximum yield of TFAD was obtained under these conditions.

### Analysis of TFAD content from different regions

TFAD was extracted from samples collected in Jimusaer, Hami, and Tacheng under optimized extraction conditions (solid-to-liquid ratio of 1:30, ethanol concentration of 50%, extraction time of 8 h, and extraction temperature of 80 °C). Four parallel replicates were conducted for each sample, with the results presented in Table 1. The relative standard deviation (RSD) values for the extraction yields of TFAD in all tested samples were less than 1.5%, confirming that the established extraction method is straightforward and exhibits excellent stability. Significant regional variation in total flavonoid content was observed. As shown in Table 2, the total flavonoid content of *A. dracunculus* from different regions of Xinjiang showed extremely significant differences (one-way ANOVA, F = 151.5658, *P* < 0.0001). The highest content was observed in Tacheng (124.8996 ± 1.3801 mg g^− 1^), followed by Hami (111.9548 ± 1.6136 mg g^− 1^), and the lowest in Jimusaer (107.0146 ± 1.4986 mg g^− 1^). Tukey’s HSD post-hoc test revealed that the total flavonoid content in Tacheng was significantly higher than that in Hami and Jimusaer (*P* < 0.0001), and the content in Hami was also significantly higher than that in Jimusaer (*P* < 0.01). Situated at 46°00′N latitude, Tacheng possesses a mid‑temperate semi‑arid climate, with a mean summer diurnal temperature range of 18–22 ℃, annual total solar radiation ranging from 5600 to 5800 MJ·m^− 2^, and annual precipitation of 270–290 mm. The optimal moisture conditions and substantial diurnal temperature fluctuations are considered the two key factors responsible for the highest flavonoid accumulation in Tacheng-grown samples.

**Table 1:** TFAD yield and corresponding RSD values of *A. dracunculus* samples from different regions of Xinjiang

Sample origin	No.	A(a.u.)	*R*(mg g^-1^)	‾$\:\stackrel{\mathrm{-}}{\mathrm{x}}$±SD	RSD
Jimusaer	1	3.203	105.2395	107.0146 ± 1.4986	1.40%
	2	3.235	106.3113		
	3	3.290	108.1534		
	4	3.296	108.3543		
Hami	1	3.336	109.6940	111.9548 ± 1.6136	1.44%
	2	3.446	113.3782		
	3	3.404	111.9715		
	4	3.428	112.7753		
Tacheng	1	3.849	126.8756	124.8996 ± 1.3801	1.05%
	2	3.756	123.7608		
	3	3.769	124.1962		
	4	3.786	124.7656		

A, absorbance (a.u.); R, TFAD yield (mg g^− 1^); RSD, relative standard deviation of TFAD yield

**Table 2:** Comparison of environmental factors in different regions

Environmental factors	Region		
	Jimusaer	Hami	Tacheng
Latitude	44°00’ N	43°36’ N	46°00’ N
Temperature range (℃)	18–22	16–18	18–22
Annual average total solar radiation(MJ/m^2^)	5400–5800	6100–6200	5600–5800
Annual average precipitation(mm)	168	167	270–290
Climate	Mid-temperate continental arid climate	Temperate cold cool arid climate	Mid-temperate continental semi-arid climate

As documented in previous research [29], large diurnal temperature variations can promote flavonoid biosynthesis by modulating the activities of key enzymes involved in plant secondary metabolism. In addition, intense ultraviolet radiation, as a typical abiotic stress factor, triggers antioxidant defense responses in plants, thereby facilitating the biosynthesis and accumulation of flavonoids. Conversely, the insignificant differences in flavonoid contents between Jimusaer and Hami samples can be attributed to their similar latitudes, nearly equivalent annual precipitation, and mutually counterbalancing variations in temperature and solar radiation.

### Evaluation of antioxidant activity of TFAD

DPPH and ABTS⁺· radicals are widely employed as classical probes in the evaluation of antioxidant activity. The ability to scavenge these radicals is considered a standard indicator of antioxidant potential. AA, a water-soluble compound with strong reducing capacity, is commonly used as a natural antioxidant reference. Its inclusion as a positive control enables direct comparison of antioxidant performance between test substances. To further assess the antioxidant activity of TFAD and examine regional variations, in vitro free radical scavenging assays were conducted. The scavenging activities of TFAD extracts from different regions and of AA were assessed against DPPH and ABTS⁺· radicals.

**Fig. 3:**
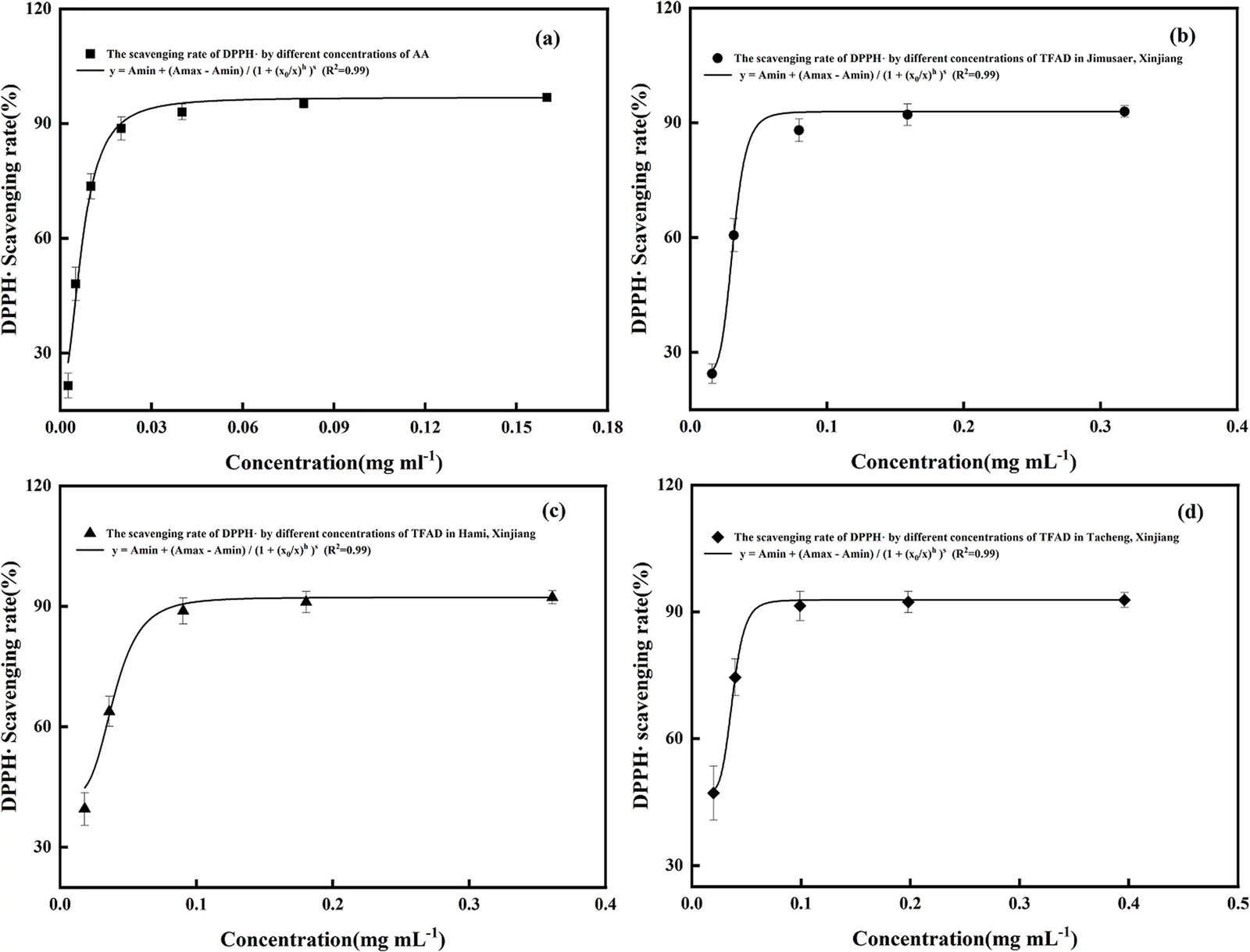
DPPH radical scavenging activity of various samples at different concentrations: **a** AA; **b** TFAD/J; **c** TFAD/H; **d** TFAD/T. DPPH: 2,2-diphenyl-1-picrylhydrazyl; AA: ascorbic acid; TFAD/J: Total flavonoids of *A. dracunculus* in Jimusaer region; TFAD/H: Total flavonoids of *A. dracunculus* in Hami region; TFAD/T: Total flavonoids of *A. dracunculus* in Tacheng region

Figure 3 presents the effects of different samples at varying concentrations on the DPPH scavenging rate. Panels (a), (b), (c), and (d) depict the scavenging activities of AA, TFAD in Jimusaer region (TFAD/J), TFAD in Hami region (TFAD/H), and TFAD in Tacheng region (TFAD/T), respectively. AA, within the concentration range of 0.00–0.18 mg mL⁻¹, and TFAD from all three regions, within the range of 0.00–0.40 mg mL⁻¹, demonstrated strong DPPH scavenging activity. A concentration-dependent increase in scavenging rate was observed across all samples. To compare the antioxidant activities of AA and TFAD from various regions, a five-parameter logistic model was employed with the asymmetry parameter fixed at 1, rendering it mathematically equivalent to the standard four-parameter logistic model (4PL). This model was applied for nonlinear regression analysis of sample concentration versus DPPH scavenging rate, and IC₅₀ values were subsequently calculated. The fitting parameters, coefficients of determination (R²), and IC₅₀ values (µg mL⁻¹) are presented in Table 3. All R² values exceeded 0.99, indicating a high degree of model fit. The IC₅₀ values for AA, TFAD/J, TFAD/H, and TFAD/T were determined to be 7.25 ± 0.56, 31.31 ± 1.19, 38.13 ± 1.56, and 37.32 ± 1.33 µg mL⁻¹, respectively. These results indicate that TFAD samples from all regions possessed measurable antioxidant activity, with significant regional variation. The order of antioxidant capacity was as follows: AA > TFAD/J > TFAD/T > TFAD/H. Among the TFAD extracts, TFAD/J exhibited the strongest scavenging activity, although it remained slightly less potent than AA. The antioxidant activity of AA was approximately 4.32 times greater than that of TFAD/J.

**Table 3:** Comparison of IC₅₀ values for DPPH· radical scavenging by different samples

Sample	Fitting parameters					
	A_min_	A_max_	x_0_(IC_50_)(µg mL ^− 1^)	h	s	*R* ^2^
AA	21.50	96.81	7.25 ± 0.56	2.29 ± 0.32	1	0.99
TFAD/J	24.4	92.92	31.31 ± 1.19	6.43 ± 3.89	1	0.99
TFAD/H	39.57	92.21	38.13 ± 1.56	3.94 ± 0.86	1	0.99
TFAD/T	47.13	92.80	37.32 ± 1.33	6.73 ± 3.70	1	0.99

*DPPH* 2,2-diphenyl-1-picrylhydrazyl, *AA* ascorbic acid, *TFAD/J* Total flavonoids of *A. dracunculus* in Jimusaer region, *TFAD/H* Total flavonoids of *A. dracunculus* in Hami region, *TFAD/T* Total flavonoids of *A. dracunculus* in Tacheng region, *A*_min_*Minimum* response value, *A*_max_ Maximum response value, *x*_0_ the half-maximal inhibitory concentration (IC_50_), *h* Hill slope, *s* asymmetry parameter

**Fig. 4:**
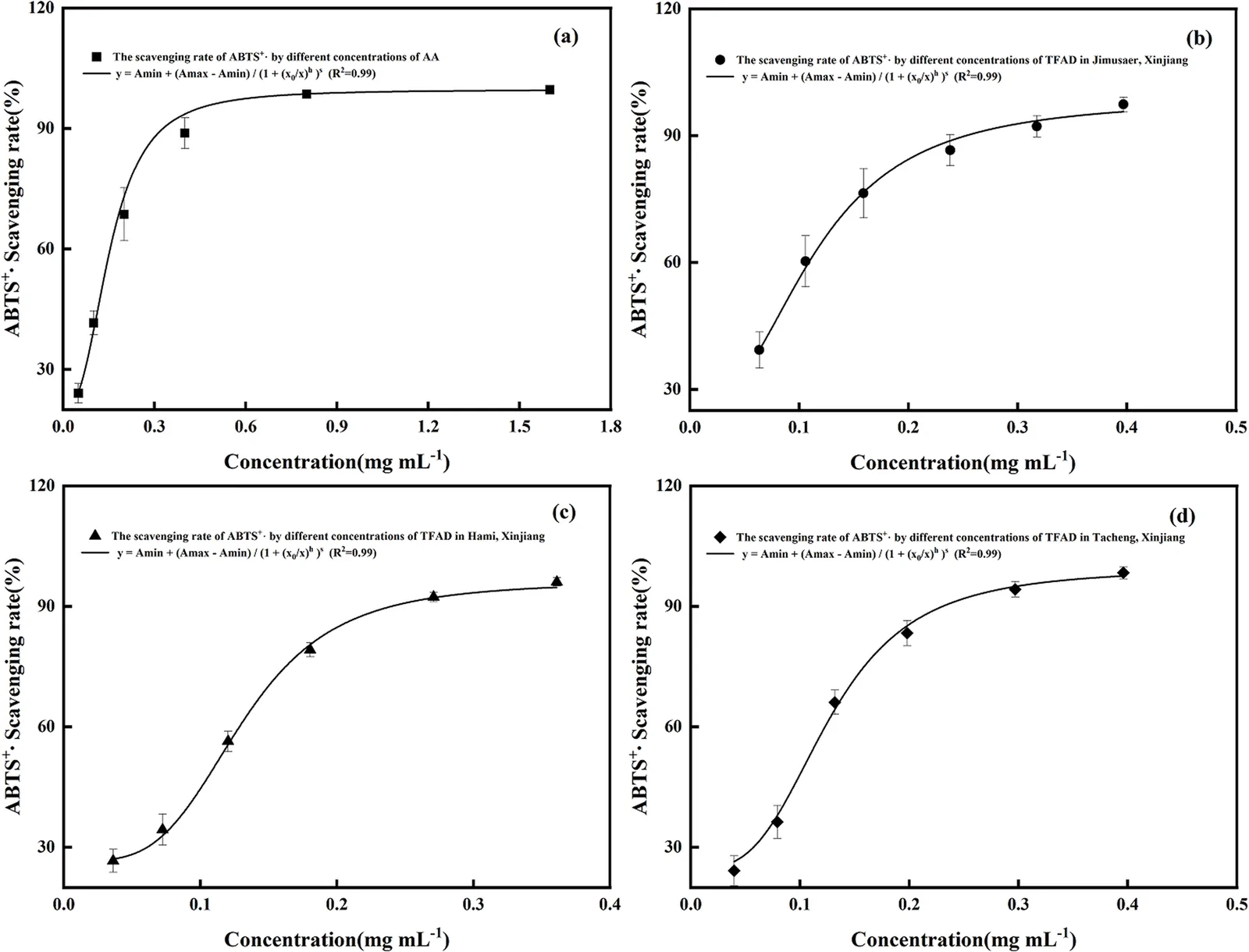
ABTS⁺· radical scavenging activity of various samples at different concentrations: **a** AA; **b** TFAD/J; **c** TFAD/H; **d** TFAD/T. ABTS⁺: 2,2′-azino-bis(3-ethylbenzothiazoline-6-sulfonic acid) radical cation; AA: ascorbic acid; Total flavonoids of *A. dracunculus* in Jimusaer region; TFAD/H: Total flavonoids of *A. dracunculus* in Hami region; TFAD/T: Total flavonoids of *A. dracunculus* in Tacheng region

Figure 4 depicts the effects of various concentrations of different samples on the ABTS⁺· scavenging rate. Panels (a), (b), (c), and (d) depict the scavenging activities of AA, TFAD from Jimusaer (TFAD/J), TFAD from Hami (TFAD/H), and TFAD from Tacheng (TFAD/T), respectively. AA (concentration range: 0.0–1.8 mg mL⁻¹) and TFAD from all three regions (concentration range: 0.0–0.4 mg mL⁻¹) demonstrated strong ABTS⁺· scavenging activity. A dose-dependent increase in scavenging rate was observed across all samples. Nonlinear fitting was conducted using the five-parameters model, as described previously. The fitted equations, coefficients of determination (R²), and IC₅₀ values (µg mL⁻¹) are presented in Table 4. All R² values reached 0.99, indicating good model performance. The IC₅₀ values of AA, TFAD/J, TFAD/H, and TFAD/T were 154.18 ± 9.54, 114.04 ± 5.56, 131.14 ± 3.08, and 127.13 ± 3.35 µg mL⁻¹, respectively, indicating substantial regional differences in antioxidant activity. The order of activity was: TFAD/J > TFAD/T > TFAD/H > AA. These results indicate that TFAD from Jimusaer, Tacheng and Hami exhibited stronger ABTS⁺· scavenging activity than AA. Notably, the antioxidant activity of TFAD/J, TFAD/T and TFAD/H was approximately 1.35-fold, 1.21-fold and 1.18-fold greater than that of AA, respectively, suggesting a potent antioxidant capacity for these three regional samples.

**Table 4:** Comparison of IC₅₀ values for ABTS⁺· radical scavenging by different samples

Sample	Fitting parameters					
	A_min_	A_max_	x_0_(IC_50_)(µg mL⁻¹)	h	s	*R* ^2^
AA	20.46	99.63	154.18 ± 9.54	2.61 ± 0.12	1	0.99
TFAD/J	25.66	98.68	114.04 ± 5.56	2.51 ± 0.19	1	0.99
TFAD/H	26.64	96.05	131.14 ± 3.08	3.88 ± 0.26	1	0.99
TFAD/T	24.85	99.30	127.13 ± 3.35	3.26 ± 0.22	1	0.99

*ABTS*⁺ 2,2′-azino-bis(3-ethylbenzothiazoline-6-sulfonic acid) radical cation, *AA* ascorbic acid, *TFAD/J* Total flavonoids of *A. dracunculus* in Jimusaer region, *TFAD/H* Total flavonoids of *A. dracunculus* in Hami region, *TFAD/T* Total flavonoids of *A. dracunculus* in Tacheng region, *A*_min_ *Minimum* response value, *A*_max_ Maximum response value, *x*_0_ the half-maximal inhibitory concentration (IC_50_), *h* Hill slope *s*: asymmetry parameter

As outlined above, *A. dracunculus* samples from Xinjiang possess high flavonoid content and strong antioxidant activity, with significant regional variation observed in both TFAD content and antioxidant capacity. To further assess the distinctiveness of Xinjiang samples and to provide a cross-regional reference for their potential development, previously published studies from other countries that explicitly reported IC₅₀ values from free radical scavenging assays were compiled and are summarized in Table 5 for comparative analysis of TFAD antioxidant activity [24, 30–32]. Although variation in reference standards among studies limits the direct comparability of absolute TFAD content across all samples, the TFAD content of the Xinjiang sample reached 107.0146 ± 1.4986 mg g⁻¹ (RSD = 1.40%), indicating a relatively high level. In terms of antioxidant activity, the IC₅₀ values for DPPH and ABTS⁺· scavenging were 31.31 ± 1.19 and 114.04 ± 5.56 µg mL⁻¹, respectively. Notably, the exceptionally low IC₅₀ value observed for ABTS⁺· scavenging highlights the strong antioxidant potential of TFAD derived from Xinjiang. The underlying mechanisms contributing to this enhanced antioxidant activity may be associated with the composition or concentration of specific bioactive constituents within the samples. However, further investigation is warranted to clarify these relationships.

**Table 5:** Comparison of TFAD content and antioxidant activity of *A. dracunculus* samples from different countries

Sample origin	*R* (mg g^-1^)	Radical species	IC_50_ (µg mL^-1^)	Reference
Xinjiang, China	107.0146 ± 1.4986 mg RE/g	DPPH·	31.31 ± 1.19	This study
		ABTS^+^·	114.04 ± 5.56	
Cluj-Napoca, Romania	181.50 ± 32.10 mg QE/g	DPPH·	69.87 ± 1.41	[24]
		-	-	
Sibiu, Romania	126.45 ± 0.32 mg QE/g	DPPH·	20.33 ± 0.2	[30]
		-	-	
Khorasan Razavi, Iran	20 ± 0.52 mg QE/g	DPPH·	65.40 ± 0.52	[31]
		-	-	
Birjand, Iran	50.4 ± 1.6 mg RE/g	DPPH·	39 ± 7	[32]
		-	-	

R, TFAD yield (mg g^− 1^); IC_50_, half-maximal inhibitory concentration (µg mL^− 1^)

*TFAD* total flavonoids extracted from *A. dracunculus*

## Conclusion

In summary, TFAD was extracted using a reflux extraction method, and the extraction parameters were systematically optimized. The results of the optimization experiments indicated that TFAD extraction efficiency is influenced by the combined effects of solvent polarity, molecular diffusion rate, and thermal degradation. Extraction temperatures exceeding 80 °C, or extraction times longer than 8 h, resulted in a decline in extraction yield, and a decline in extraction yield was observed under extreme conditions such as ethanol concentration greater than 60%. The highest extraction efficiency was achieved using 50% ethanol, a solid-to-liquid ratio of 1:30, an extraction temperature of 80 °C, and a duration of 8 h. The optimized method was found to be straightforward, efficient, and reproducible. Total flavonoids were extracted from *A. dracunculus* samples collected in Jimusaer, Hami, and Tacheng, Xinjiang using these optimized conditions. The TFAD content followed the order: TFAD/T > TFAD/H > TFAD/J, with statistically significant regional differences. The highest flavonoid content was observed in the sample from Tacheng, reaching 124.8996 ± 1.3801 mg g⁻¹ (RSD = 1.05%). Based on the distinct environmental characteristics of the Tacheng region and findings reported in previous studies, it is hypothesized that the combination of high ultraviolet radiation, large diurnal temperature variation and relatively sufficient moisture may contribute to the enhanced biosynthesis and accumulation of flavonoid compounds in *A. dracunculus* grown in this area.

The antioxidant activity of TFAD extracted from different regions of Xinjiang was assessed through free radical scavenging assays and compared with reported antioxidant activities of TFAD obtained from *A. dracunculus* samples collected in Romania and Iran. The results demonstrated that TFAD from Xinjiang exhibited notably stronger antioxidant activity. In the DPPH and ABTS⁺· scavenging assays, the antioxidant activity of TFAD from different regions of Xinjiang followed the orders: AA > TFAD/J > TFAD/T > TFAD/H and TFAD/J > TFAD/T > TFAD/H > AA, respectively. Comparative analysis indicated that the ranking of antioxidant activity did not correspond to the ranking of total flavonoid content, a finding consistent with previous reports. This suggests that antioxidant activity is not solely dependent on the overall flavonoid concentration but may be more closely related to the composition or relative abundance of specific bioactive constituents. These results highlight a potential direction for future investigation into the specific compounds responsible for antioxidant activity.

Among the samples analyzed, TFAD from Jimusaer (TFAD/J) demonstrated the highest antioxidant activity in both DPPH and ABTS⁺· scavenging assays, with IC₅₀ values of 31.31 ± 1.19 and 114.04 ± 5.56 µg mL⁻¹, respectively. In the ABTS⁺· assay, the antioxidant activity of TFAD/J was 1.35-fold greater than that of AA, indicating a significantly enhanced radical scavenging capacity. The combination of natural origin, high antioxidant efficiency, and low toxicity suggests that TFAD/J may hold potential for application in the food, pharmaceutical, and personal care industries.

It should be emphasized that neither in vitro nor in vivo activity validation was performed in the present study. Limited by the experiment, the lower asymptote of the fitted curve in the free radical scavenging experiment could not be anchored at 0%. The IC_50_ value reported in this study is a calculated value based on the current concentration range in this research. Additionally, chromatographic characterization of the flavonoid composition—which could help explain the observed bioactivity differences among regions—was not feasible due to the limited availability of commercial standards specific to *A. dracunculus*. These important aspects—bioactivity validation and flavonoid composition analysis—will be systematically addressed in our future research.

## Data Availability

All data generated or analysed during this study are included in this article. Further enquiries can be directed to the corresponding author.

## Abbreviations

TFAD: Total flavonoids from A. dracunculus
AA: Ascorbic Acid
DPPH: 2,2-Diphenyl-1-picrylhydrazyl
ABTS: 2,2’-Azinobis-(3-ethylbenzthiazoline-6-sulphonate)
ROS: Reactive Oxygen Species
DNA: Deoxyribonucleic acid
IC_50_: Half maximal inhibitory concentration
RSD: Relative standard deviation
10.R^2^: R-squared
TFAD/J: Total flavonoids of A. dracunculus in Jimusaer region
TFAD/H: Total flavonoids of A. dracunculus in Hami region
TFAD/T: Total flavonoids of A. dracunculus in Tacheng region
